# A Computational Study of the Heterogeneous Synthesis of Hydrazine on Co_3_Mo_3_N

**DOI:** 10.1007/s10562-017-2080-y

**Published:** 2017-05-24

**Authors:** Constantinos D. Zeinalipour-Yazdi, C. Richard A. Catlow

**Affiliations:** 1 Kathleen Lonsdale Materials Chemistry, Department of Chemistry, University College London, London, WC1H 0AJ UK; 2 School of Chemistry, Cardiff University, Cardiff, CF10 1AD UK

## Abstract

**Abstract:**

Periodic and molecular density functional theory calculations have been applied to elucidate the associative mechanism for hydrazine and ammonia synthesis in the gas phase and hydrazine formation on Co_3_Mo_3_N. We find that there are two activation barriers for the associative gas phase mechanism with barriers of 730 and 658 kJ/mol, corresponding to a hydrogenation step from N_2_ to NNH_2_ and H_2_NNH_2_ to H_3_NNH_3_, respectively. The second step of the mechanism is barrierless and an important intermediate, NNH_2_, can also readily form on Co_3_Mo_3_N surfaces via the Eley–Rideal chemisorption of H_2_ on a pre-adsorbed N_2_ at nitrogen vacancies. Based on this intermediate a new heterogeneous mechanism for hydrazine synthesis is studied. The highest relative barrier for this heterogeneous catalysed process is 213 kJ/mol for Co_3_Mo_3_N containing nitrogen vacancies, clearly pointing towards a low-energy process for the synthesis of hydrazine via a heterogeneous catalysis route.

**Graphical Abstract:**

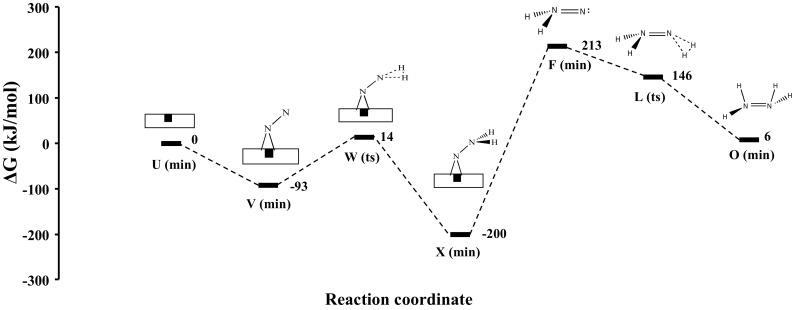

**Electronic supplementary material:**

The online version of this article (doi:10.1007/s10562-017-2080-y) contains supplementary material, which is available to authorized users.

## Introduction

The development of alternative routes to convert N_2_ to ammonia (NH_3_) and hydrazine (H_2_NNH_2_) could have significant economic and environmental impact, since more that 50% of ammonia for soil fertilisers is produced industrially and the global annual production of hydrazine is more than 80 thousand tons [1]. Ammonia is industrially produced mostly via the classical Haber–Bosch (H–B) process [2–4], by a Fe–K_2_O–Al_2_O_3_ catalyst under high temperatures (>400 °C) and pressures (150–200 atm). Some production has shifted towards the Kellogg advanced ammonia process, which uses a graphite-supported alkali/alkaline-earth promoted Ru catalyst and operates at milder conditions [5].

Hydrazine is produced by the Bayern ketazine process, which is a variation of the Olin Raschig process [6]. The two main reactions of this process are the formation and hydrolysis of 1,2-di(propan-2-ylidene)hydrazine (i.e. acetone azine),

$${2M}{{e}_{2}}{CO }+{ 2N}{{H}_{3}}+{ NaOCl } \to {M}{{e}_{2}}{C}={NN}={CM}{{e}_{2}}+{ 3}{{H}_{2}}{O }+{ NaCl},$$

$${M}{{e}_{2}}{C}={NN}={CM}{{e}_{2}}+{ 2}{{H}_{2}}{O } \to { }{{N}_{2}}{{H}_{4}}+{ 2M}{{e}_{2}}{CO}.$$

In brief, in this process ammonia is oxidised by sodium hypochlorite in acetone to form acetone azine (ketazine) which in the presence of water decomposes to hydrazine and acetone. A by product of the reaction is sodium chloride. The details of this process are: sodium hypochlorite (NaOCl) solution, ammonia (NH_3_) and acetone (Me_2_CO) are mixed at 35 °C. A solution results consisting of about 7% by weight acetone azine together with sodium chloride and excess ammonia. Ammonia is distilled off and returned to the reaction. The acetone azine-water-azeotrope (b.p. 95 °C) is distilled off leaving the sodium chloride solution. The acetone azine is hydrolyzed with water at temperatures up to 180 °C and pressures of 8–12 bar into an acetone and an aqueous hydrazine solution. The hydrazine solution is then concentrated by distillation.

Co_3_Mo_3_N is one of the most active catalysts for ammonia synthesis at mild conditions, especially when doped with caesium [7–11]. In two recent density functional theory (DFT) studies we have identified possible sites for the adsorption and activation of the reactants of the ammonia synthesis reaction on a model Co_3_Mo_3_N surface, with heterogeneity due to surface nitrogen vacancies [12]. Such vacancies are present in large concentrations even at ambient temperatures (i.e. ~10^13^ cm^−2^) and can efficiently activate N_2_ [13]. In particular, we found that N_2_ can be activated at a surface cavity where N_2_ is bound *side-on* a Co_8_ cluster at the *16e* Wyckoff site or it can be activated at surface 3f-nitrogen vacancies where N_2_ is activated in an *end-on* configuration [12]. There are other DFT studies that have shown that N-vacancies can participate in the mechanism for ammonia synthesis, such as in the electrochemical reduction of ammonia on Zr, Nb, Cr, V mononitrides [14–16] and in the two-phase solar-energy driven ammonia synthesis on metal-nitrides [17, 18]. *End-on* activated N_2_ also exists in Fe(0) complexes (i.e. Fe(Et_2_PCH_2_CH_2_PEt_2_)_2_(N_2_)) found to be selective for the formation of hydrazine from N_2_ with NH_3_ forming at a ratio of 25:1 [19]. These complexes were initially designed to resemble the binding of N_2_ to FeMo-cofactor [20], the enzyme responsible for the biological fixation of nitrogen in nitrogenases, for which further atomistic details on the mechanism have recently been reported [21].

To assist our understanding of the hydrazine synthesis reaction, we have first modelled the reaction mechanism for associative synthesis of hydrazine and ammonia in the gas phase, via hybrid DFT calculations and also modelled how some of the intermediates can be formed on the surface of Co_3_Mo_3_N. The results of this paper are presented in the following order. First we present the various hydrogenated intermediates of the hydrazine and ammonia synthesis reaction in the gas phase. Then we present a gas-phase, free energy diagram that explores the kinetic pathway for hydrazine and ammonia synthesis. Lastly we present a new mechanism that shows how hydrazine could be synthesized over a heterogeneous catalyst (i.e. Co_3_Mo_3_N).

## Methods and Models

Molecular open and closed-shell DFT computations have been perfomed with Gaussian 09 [22] with the use of Becke’s three-parameter hybrid exchange functional [23] (XC) combined with the Lee–Yang–Parr non-local correlation functional [24], abbreviated as B3LYP. For the basis functions we have used the spherical version (i.e. 5d, 7f), the correlation consistent augmented valence triple zeta basis set [25–29], abbreviated as aug-cc-pVTZ.

The potential free energy diagrams were obtained by plotting the formation Gibbs free energy change ${({\Delta} G}_{f,PES})$ of the various intermediates and transitions states with respect to gas phase reactants, given by the following expression,

1 $$\Delta {G_{f,PES}}={G_{NxHy}} - \frac{x}{2} \cdot {G_{N2}} - \frac{y}{2} \cdot {G_{H2}}$$

In the computed free energy changes we have included the entropy changes for gas phase molecules the vibrational energy of the adsorbates bound to a single metal atom, therefore entropy changes for the solid phase have been omitted. These were calculated at P = 1 atm and T = 298.15 K. The equation from which the vibrational energies of the adsorbates was calculated is given in the supporting information file. Intermediates and transition states (TS) have been confirmed using vibrational analysis, by the absence and presence of one imaginary vibrational frequency, respectively. Transition state structures were either located using the Synchronous Transit-Guided Quasi-Newton (STQN) method of Schlegel and co-workers [30, 31] or by scanning a particular bond length at a 0.05 Å resolution and relaxing the remaining atoms. The imaginary frequency of the located TS was animated to ensure that it corresponds to the desired reaction coordinate. Every structure was calculated at a spin multiplicity (s.m.) of 1, 3 and 5, but only the lowest energy structure is reported. Calculations were checked for spin contamination, which was found to be negligible. In most cases, the structure with s.m. = 1 had the lowest energy. The molecular structure, point group (p.g.) symmetry, vibrational streching frequency (ν_N−N_), bond length (r_N−N_), spin multiplicity and formation energy (ΔG_f_) of the various N_x_H_y_ intermediates are shown in Table 1. The table is partioned to show the various stable hydrogenation intermediates N_x_H_y_ that were found. These can assist infrared spectroscopic efforts in identifying the intermediates in the gas phase reaction mechanism. The intermediate of interest, **F**, could be identified through IR spectroscopy as its ν_N−N_ differs by more than 400 cm^−1^ from that of isomeric **G, H** and **I**. Furthermore, Table 1 lists the computed free energies of formation of the various intermediates at P = 1 atm and T = 298.15 K. These formation energies show the relative trends of the stability of the various intermediates of the same molecular weight and are therefore useful in the experimental isolation of such intermediates during the synthesis of ammonia and hydrazine. We find good agreement between the computed formation free energy of ammonia −23 kJ/mol with the one listed in thermeodynamic tables (i.e. CRC Handbook of Chemistry and Physics) −27 kJ/mol, indicative that a relatively accurate computational methodology has been followed.

**Table 1:** Calculated at B3LYP/aug-cc-pVTZ properties of the various N_x_H_y_ intermediates participating in the gas phase mechanism of ammonia synthesis

	Formula	p.g.	State	v_N−N_ (cm^−1^)	r_N−N_ (Å)	s.m.	ΔG_f_ (kJ/mol)
A		D_infh_	Min	2448	1.091	1	–
B		D_infh_	Min	[4417]	[0.7429]	1	–
C		C_2v_	TS	719	1.433	3	886
D		D_2h_	TS	954	1.467	1	768
E		C_s_	TS	738	1.433	1	730
F		C_s_	Min	1208	1.336	3	352
G		C_2v_	Min	1608	1.210	1	300
H		C_2v_	Min	1647	1.234	1	236
I		C_2h_	Min	1652	1.235	1	215
J		C_2_	TS	1136	1.429	1	746
K		C_2v_	TS	1073	1.436	1	346
L		C_s_	TS	1045	1.468	1	285
M		C_s_	Min	1434	1.314	3	474
N		C_2v_	TS	909.2	1.477	3	179
O		C_2_	Min	1107	1.433	1	145
P		C_2_	TS	571	1.600	1	892
Q		C_1_	TS	649	1.467	3	837
R		D_3d_	Min	1032	1.464	1	632
S		D_3_	Min	392	2.218	3	399
T		C_3v_	Min	–	–	1	−23

The parameters listed are the point group (p.g.) symmetry, the state, vibrational streching frequency (ν_N−N_), bond length (r_N−N_), spin multiplicity (s.m.) and formation energy (ΔG_f_) of the various N_x_H_y_ intermediates. P = 1 atm and T = 298.15 K. Values in brackets refer to the H–H bond

Periodic calculations were performed with the VASP code with a 650 eV cutoff for the planewave expansion and the revPBE XC functional. A 4 × 4 × 1 Γ-point centered MP grid for the 2 × 2 surface supercell with one 3f-hollow nitrogen vacancy and a vacuum gap of 20 Å was used. All calculations were spin-polarised.

## Results and Discussion

### Hydrazine and Ammonia Synthesis in Gas Phase

**Fig. 1:**
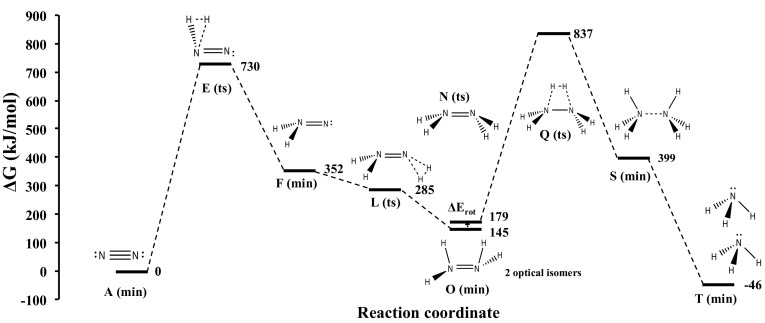
Gibbs free energy diagram for associative hydrazine and ammonia synthesis mechanism in gas phase calculated at B3LYP/aug-cc-pVTZ (5d, 7f). Free energies of formation of the various intermediates and transition states have been calculated using Eq. 1 and are tabulated in Table 1 at P = 1 atm and T = 298.15 K

We have studied the associative mechanism for ammonia and hydrazine synthesis, where N_2_ does not dissociate but rather reacts directly with H_2_, which necessitates very high pressure rather than thermal activation of the reactants. This mechanism of ammonia synthesis in the gas phase can be directly compared to the associative mechanism occurring on the (111) surface of Co_3_Mo_3_N. The resulting free energy diagram for the associative mechanism of ammonia synthesis in the gas phase is shown in Fig. 1, where we only present the kinetic pathway (the pathway which has the lowest barrier for its rate-determining step (RDS)). We note that there is also another mechanism which proceeds via monotamic hydrogen atoms and provides an energetically viable alternative as a result of the high activation barrier of the first hydrogenation step (730 kJ/mol) for the associative mechanism which based on the bond-dissociation enthalpy (BDE) of H_2_ would cause the H–H bond to break before the formation of intermediate N_2_H_2_ (**F)**. However, we only consider the associative mechanism due to its relevance to the reaction occurring on the Co_3_Mo_3_N surface. We find that for the gas phase reaction, the various mechanistic pathways generally consist of three activated steps, where each step corresponds to a hydrogenation reaction of H_2_ with N_2_ forming N_2_(H_2_)_n_ (n = 0, 1, 2) intermediates. For the kinetic pathway only the first and the last hydrogenation steps have considerably high barriers, whereas in the second the barrier was essentially absent. The kinetic pathway was determined on the basis that its RDS had the lowest barrier among the four possible associative pathways studied. We find that the RDS step for ammonia synthesis in the gas phase (without a catalyst) has a barrier of 730 kJ/mol, which is followed by a barrier of 658 kJ/mol which corresponds to the third hydrogenation step. From the relative barrier heights of the 1st and 3rd hydrogenation steps in Fig. 1 it is suggested that based on an associative mechanism, there would be some hydrazine formation during ammonia synthesis. This has been previously observed in glow discharge reactions of N_2_ and H_2_ over various solid surfaces (e.g. SiO_2_, TiO_2_, MoO_3_, Al_2_O_3_, MgO, Mg(OH)_2_) having a zeroth order dependence on the reactants total pressure, producing 4–28% hydrazine and 72–96% ammonia [37]. The 658 kJ/mol barrier could be overcome if the **O** isomer of hydrazine radiatively undergoes isomerization through intermediate **N** (see Table 1), which brings the hydrazine intermediate **N** to the right stereochemical structure to undergo subsequent hydrogenation to form H_3_NNH_3_ (**S**). We have calculated that this requires far-infrared radiation of a wavelength of 2842 cm^−1^. **S** thermally decomposes to form two ammonia molecules in a barrierless process. The intriguing aspect of this mechanism is that the 2nd hydrogenation step is barrierless, which corresponds to the adsorption of molecular hydrogen on the hydrogen pre-activated intermediate N_2_H_2_ (**F**). Interestingly we also found that this intermediate can form readily on (111)-surfaces of Co_3_Mo_3_N, at nitrogen vacancy sites. This intermediate in the gas phase reaction mechanism requires 730 kJ/mol to form but only 107 kJ/mol at the nitrogen vacancies on Co_3_Mo_3_N. We seek this intermediate and the mechanism for hydrazine synthesis in the associative mechanism for ammonia synthesis in the following section.

### Hydrazine Synthesis on Co_3_Mo_3_N

**Fig. 2:**
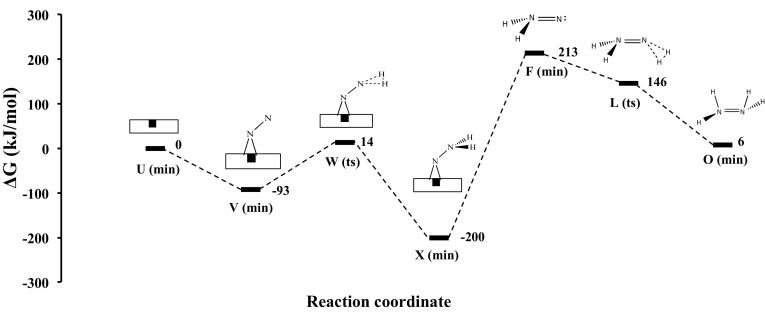
Gibbs free energy diagram of heterogeneous mechanism occurring on Co_3_Mo_3_N in the presence of nitrogen vacancies (*black square*). XC: revPBE (650 eV) for the surface mechanism and B3LYP/aug-cc-pVTZ for the gas phase steps. The choice of these XC functionals is justified based on the mean-average-percent-error that various GGA and hybrid-GGA functionals have in evaluating bond-dissociation enthalpies in the supporting information of Ref. [12]. P = 1 atm and T = 298.15 K. Structures of the surface reactions on Co_3_Mo_3_N are shown in Fig. 3

In this study we have found that intermediate **F** on Co_3_Mo_3_N can form at nitrogen vacancy sites, similar to its formation on Mo = NNH_2_ [32] and Fe-NNH_2_ [33] complexes and in a DFT study of the Schrock cycle molybdenum complex [Mo(hiptN_3_N)] [34]. It is noted that this activation site of Co_3_Mo_3_N shows some similarities with the FeMo-cofactor with the main difference the bridging-N rather than bridging-S sites. A detailed study of the various adsorption sites on Co_3_Mo_3_N surfaces is given in Ref. [13]. The D3-corrected adsorption energy of N_2_ is moderately exothermic, evidence that the adsorption of N_2_ at the nitrogen vacancies can occur at ambient temperatures. N_2_ in this position is activated by 11%, based the adsorption induced N–N bond lengthening. Once N_2_ is adsorbed to the surface in an end-on configuration it reacts with H_2_ coming from the gas phase by an activated step of only 107 kJ/mol in order to form intermediate **X**. The bonding of intermediate **X** resembles intermediate **L** in the gas phase mechanism. The optimal molar ratio of N_2_:H_2_ in the gas feed-stream is found to be 1:1, in order manifest N_2_ adsorption at nitrogen vacancies and H_2_ co-adsorption at Co_8_ clusters [13]. This prohibits poisoning of the nitrogen activation sites by chemisorbed hydrogen although we have previously shown via DFT that even at ambient T, there are more than 10^13^ nitrogen vacancies per cm^2^ which can act as active sites for nitrogen activation [12]. The energy required to generate nitrogen vacancies, the vacancy formation energy (VFE) was found to be 162 kJ/mol for three-fold (3f) bound nitrogen. This clearly suggests that if the reaction is run with a net input of energy of 213 kJ/mol (see barrier for desorption of intermediate **X** from the nitrogen vacancy in Fig. 2) then nitrogen activation sites will constantly be present and replenished even if some hydrogen adsorption occurs at these sites, whose adsorption is generally more exothermic than nitrogen chemisorption. Once intermediate **X** is formed on the catalyst surface, from the free energy diagram shown in Fig. 2, we see that the energy required to achieve its desorption is relatively high but still much lower than the barrier of the gas phase mechanism (730 kJ/mol). Therefore some decomposition of intermediate **X** back to the reactants N_2_ and H_2_, should be expected as this is observed also over MoN_x_/γ–Al_2_O_3_ [35] and activated carbon supported tungsten carbide [36]. Once NNH_2_ is in the gas phase (i.e. intermediate **F**, a small desorption barrier has been ignored) it reacts in a barrierless process with H_2_(g) as shown in Fig. 2. It is noted that NNH_2_ can also undergo surface hydrogenation reactions which lead to the formation of ammonia rather then hydrazine. Through this newly suggested mechanism the synthesis of hydrazine is possible on Co_3_Mo_3_N with a relative barrier of the RDS of only 213 kJ/mol, which is 517 kJ/mol lower than the gas phase associative mechanism for hydrazine synthesis. It is therefore suggested that this Eley–Rideal synthesis of hydrazine should be tested under the conditions described with the Co_3_Mo_3_N catalyst.

**Fig. 3:**
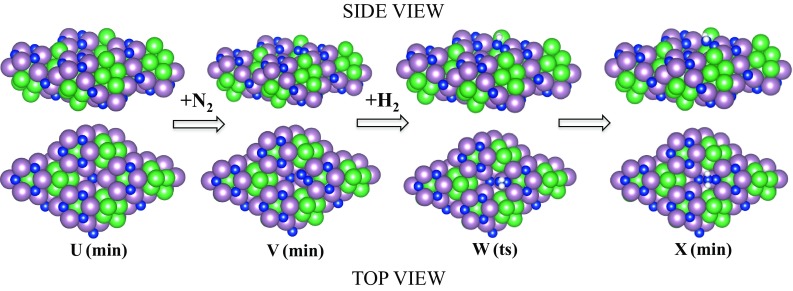
Sphere-in-contact models of the Eley–Rideal surface reaction of N_2_ and H_2_ on Co_3_Mo_3_N in the presence of a nitrogen vacancy. Nitrogen: *blue*, cobalt: *green*, molybdenum: *pink* and hydrogen: *white*

To summarise, our DFT study shows that hydrazine can be formed with relatively small energy input (213 kJ/mol) on Co_3_Mo_3_N-(111) surfaces, compared to the gas phase mechanism which requires 730 kJ/mol for the associative mechanism. The free energy diagrams presented are critical for the design of new experiments to show the possibility of hydrazine synthesis from N_2_ and H_2_. The infrared absorption frequencies presented of the various ammonia synthesis intermediates are necessary for spectroscopic identification of the reaction intermediates. We therefore anticipate that this study will initiate experimental efforts to establish a heterogeneous catalytic route for the synthesis hydrazine.

## Electronic supplementary material

Below is the link to the electronic supplementary material.

Supplementary material 1 (DOCX 27 KB)
