# Supplementary material for: A Computational Study of the Heterogeneous Synthesis of Hydrazine on Co3Mo3N
Source: Catal Letters. 2017 May 24;147(7):1820–6. doi: 10.1007/s10562-017-2080-y (PMC6979644; doi:10.1007/s10562-017-2080-y)
Supplement: Supplementary file 1 — Supplementary material 1 (DOCX 27 KB) [file 10562_2017_2080_MOESM1_ESM.docx]

# *supporting information*

# A computational study of the heterogeneous synthesis of hydrazine on Co_3_Mo_3_N

Constantinos D. Zeinalipour-Yazdi^1,*^ and C. Richard A. Catlow^1,2*^

*^1^ Kathleen Lonsdale Materials Chemistry, Department of Chemistry, University College London, London, WC1H 0AJ, UK*

*^2^ School of Chemistry, Cardiff University, Cardiff CF10 1AD, UK*

The free energy of the various adsorbates relative to the gas phase molecular N_2_ and H_2_ was obtained from the expression

$$\Delta G=\Delta E+\Delta E_{ZPV}-T\Delta S$$

where $E_{ZPV}$, the zero-point vibrational energy was calculated for the corresponding molecular cluster models via the B3LYP/aug-cc-pVDZ method in order to accurate obtain the oscillator frequencies of the various adsorbed intermediates, given by

$$E_{ZPV}=\sum_{i} \frac{1}{2}h\nu_{i}$$

whereas the vibrational entropy was calculated from the statistical mechanical expression

$$S=R\sum_{i} \left( \frac{\frac{h\nu_{i}}{k_{B}T}}{exp\left( \frac{h\nu_{i}}{k_{B}T} \right)-1}-ln\left[ 1-exp\left( -\frac{h\nu_{i}}{k_{B}T} \right) \right] \right)$$

where *R* is the gas constant, *h* Planck's constant, $k_{B}$ Boltzmann's constant, *T* the absolute temperature and $\nu_{i}$ the ith vibrational frequency given. Rotational and translational entropies were assumed to be negligible compared to the vibrational entropy for all surface intermediates, except for the reactansts (i.e. N_2_ and H_2_) and products (i.e. NH_3_) molecules that are in the gas phase.
